# Cloning and biochemical characterization of an endo-1,4-β-mannanase from the coffee berry borer *hypothenemus hampei*

**DOI:** 10.1186/1756-0500-6-333

**Published:** 2013-08-22

**Authors:** Carolina Aguilera-Gálvez, Juan J Vásquez-Ospina, Pablo Gutiérrez-Sanchez, Ricardo Acuña-Zornosa

**Affiliations:** 1 Biología de la Conservación y Biotecnología UNISARC. Facultad de Ciencias Básicas, Corporación Universitaria Santa Rosa de Cabal, Km 4 vía antigua Santa Rosa de Cabal, Chinchiná, Colombia; 2 Disciplina de Mejoramiento Genético, Centro Nacional de Investigaciones de Café (CENICAFE), Planalto, Km 4 vía antigua, Chinchiná-Manizales, Colombia; 3 Laboratorio de Microbiología Industrial, Facultad de Ciencias, Universidad Nacional de Colombia, Sede Medellín, Colombia

**Keywords:** Endo-mannanase, Coffee berry borer, *Hypotenemus hampei*, Glycosyl hydrolase

## Abstract

**Background:**

The study of coffee polysaccharides-degrading enzymes from the coffee berry borer *Hypothenemus hampei,* has become an important alternative in the identification for enzymatic inhibitors that can be used as an alternative control of this dangerous insect. We report the cloning, expression and biochemical characterization of a mannanase gene that was identified in the midgut of the coffee berry borer and is responsible for the degradation of the most abundant polysaccharide in the coffee bean.

**Methods:**

The amino acid sequence of HhMan was analyzed by multiple sequence alignment comparisons with BLAST (Basic Local Alignment Search Tool) and CLUSTALW. A *Pichia pastoris* expression system was used to express the recombinant form of the enzyme. The mannanase activity was quantified by the 3,5-dinitrosalicylic (DNS) and the hydrolitic properties were detected by TLC.

**Results:**

An endo-1,4-β-mannanase from the digestive tract of the insect *Hypothenemus hampei* was cloned and expressed as a recombinant protein in the *Pichia pastoris* system. This enzyme is 56% identical to the sequence of an endo-β-mannanase from *Bacillus circulans* that belongs to the glycosyl hydrolase 5 (GH5) family. The purified recombinant protein (rHhMan) exhibited a single band (35.5 kDa) by SDS-PAGE, and its activity was confirmed by zymography. rHhMan displays optimal activity levels at pH 5.5 and 30°C and can hydrolyze galactomannans of varying mannose:galactose ratios, suggesting that the enzymatic activity is independent of the presence of side chains such as galactose residues. The enzyme cannot hydrolyze manno-oligosaccharides such as mannobiose and mannotriose; however, it can degrade mannotetraose, likely through a transglycosylation reaction. The K_m_ and k_cat_ values of this enzyme on guar gum were 2.074 mg ml^-1^ and 50.87 s^-1^, respectively, which is similar to other mannanases.

**Conclusion:**

This work is the first study of an endo-1,4-β-mannanase from an insect using this expression system. Due to this enzyme’s importance in the digestive processes of the coffee berry borer, this study may enable the design of inhibitors against endo-1,4-β-mannanase to decrease the economic losses stemming from this insect.

## Background

As an extension of previous study on different glycosyl hydrolase (GHs) enzymes that are secreted in the digestive system from coffee berry borer *Hypothenemus hampei,* this investigation is concerned with an specific GHs, an endo-1,4-β-mannanase (EC 3.2.1.78) [1]. This enzyme plays an important role in the random internal hydrolysis of β-mannosidic links in the main structure of mannans, releasing small manno-oligosaccharides (MOS) that are absorbed by the berry borer and used for development and growth. Thus, mannanases are an important inhibitory target in the design of control strategies for the coffee berry borer. Previous studies have relied in synthetic inhibitors evaluation of this enzyme [2].

Mannans are a group of polysaccharides that include four types: linear mannans, galactomannans, glucomannans and galactoglucomannans [3]. These polysaccharides are structural components of the cell walls and intracellular matrices of terrestrial and marine plants. In the coffee bean (*Coffea sp.*), a crop that provides sustenance to approximately half a million families of growers in Colombia [4], galactomannans constitute 25% of total polysaccharides, which in turn represents 50% of the coffee bean’s dry weight [5].

This seed is the only food source for the coffee berry borer (*Hypothenemus hampei*), which is the most detrimental insect for coffee growers around the world [6]. Throughout its life cycle, the berry borer remains inside the fruit, thus making it difficult to control with insecticides or natural enemies. Searching for mechanisms that interfere with the insect’s normal physiology may be the foundation for developing alternative biotechnologies for controlling this insect population.

Other studies have focused on bacterial endo-1,4-β-mannanases [7-9], fungi [10-12], superior plants [13] and mollusks [14,15]. In this article, the cloning and biochemical characterization of a recombinant endo-1,4-β-mannanase (rHhMan) is described for the first time for the *H. hampei* insect. Additionally, the investigation of this enzyme’s hydrolytic properties allows characteristics associated with the mechanism of this enzyme to be inferred to facilitate the design of inhibitors for use in future control strategies.

## Results and discussion

### Sequence analysis of the HhMan

**Figure 1:**
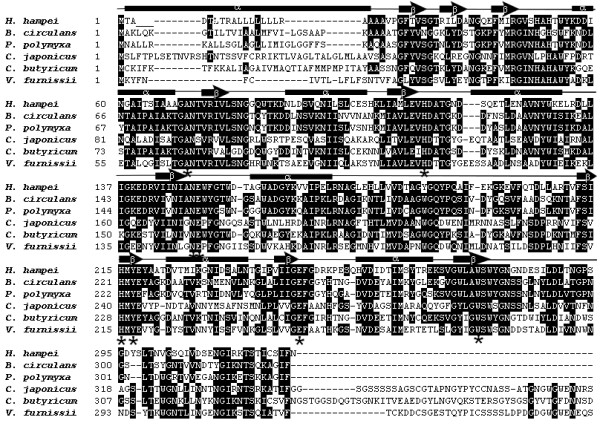
**Alignment of the deduced amino acid sequence of HhMan of *Hypothenemus hampei*** **with those of other GH5 mannanases.** Identical or similar amino acid residues are listed in black or gray, respectively. Glutamic acid residues and the residues highly conserved of the active site are indicated asterisk (*) below the alignment. The regions of HhMan with secondary structure α-helices or β-folded sheets shown on the alignment.

Pfam analysis (E-value: 1.4×10^-34^) indicated that the enzyme belongs to the glycosyl hydrolase GH5 family, an extensive group of enzymes that catalyze the cleavage of glycosidic bonds between two or more carbohydrates [16,17]. These results were demonstrated by multiple alignment of the deduced amino acid sequence against other mannanases of distinct origins within this family: *Bacillus circulans* (GenBank Accession Number AAX87003.1), *Paenibacillus polymyxa* (GenBank Accession Number ADO54643.1), *Cellvibrio japonicus* (GenBank Accession Number AA031760.1), *Clostridium butyricum* (GenBank Accession Number EEP53331.1) and *Vibrio furnissii* (GenBank Accession Number EEX39836.1), yielding percentage identities (PID) of 56%, 57%, 39%, 52% and 39% respectively. Figure 1 shows that all of the mannanases in the analysis exhibit features common to the GH5 family, including two strictly conserved glutamic acid residues that are important for catalytic activity (E147 and E242) by acting as nucleophiles or general acid/base catalysts, as well as six active site catalytic residues (R72, H108, N146, H212, Y214 and W272) [18-21].

### Cloning and expression of the *HhMan* gene

The *HhMan* gene was amplified by PCR with primers designed from the available GenBank sequence (ADF22325.1) and cloned in the pPICZαA vector. The coding sequence corresponds to a 900-bp open reading frame (ORF) for a 300-aa protein and does not include the native signaling sequence.

**Figure 2:**
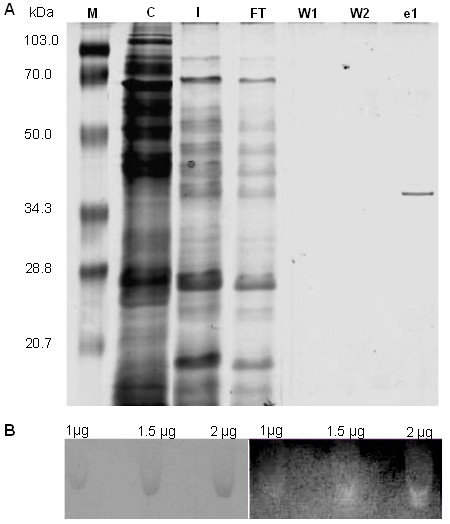
**SDS- page, native- page and zymogram of the purified rHhMan. A)** SDS-PAGE. M: Broad Range weight marker, C: Negative control for expression, I: Unpurified samples. FT: Fraction not retained in the matrix. W1: wash 1, W2: wash 2, e1: purified rHhMan. **B)** NATIVE- PAGE AND ZYMOGRAM Samples of pure protein (1, 1.5 y 2μg) were loaded on two native gels (12%). Left panel in absence of guar gum, the gel was stained with Coomassie brilliant blue R250 to visualize the band corresponing to mannanase (35.5 kDa). Right panel in the presence of 1% guar gum, the gel was stained with Congo red to verify the mannanase activity.

rHhMan was expressed as an extracellular protein in *Pichia pastoris.* The protein content in the supernatant was purified by affinity chromatography on Ni-NTA agarose with a one-step purification protocol. SDS-PAGE analysis demonstrated that an apparently homogeneous protein was obtained with an approximate molecular weight of 35.5 kDa, corresponding to 33 kDa from the protein sequence without the native signaling sequence and 2.5 kDa from the N-terminal polyhistidine tag (Figure 2A). The calculated molecular weight range for rHhMan (35–40 kDa) lies within those previously reported for other mannanases, such as mannanases from *Solanum lycopersicon* (39 kDa) [22], *Bacillus subtilis* (39.6 kDa) [9], *Aplysia kurodai* (40 kDa) [23] and *Cryptopygus antarcticus* (40 kDa) [24].

The expression protocol yielded 6 mg of pure protein per liter of culture medium with a specific activity on guar gum of 1075.3 U/mg under standard assay conditions.

Enzyme activity of the commercial enzyme was checked (data not shown). The enzymatic activity of rHhMan was evaluated by zymography assays in 1% guar gum substrate. The enzymatic activity was directly proportional to the enzyme concentration. Figure 2B shows that the highest activity levels were obtained with 2 μg of enzyme, which was the highest concentration tested. The activity was reduced when a lower concentration of 1 μg was used.

### Effects of pH and temperature on enzymatic activity

**Figure 3:**
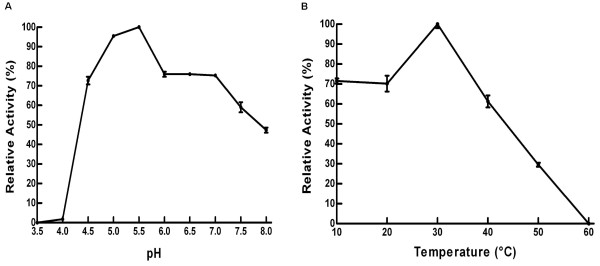
**Characterization of recombinant rHhMan. A) **The effect of pH on the activity of rHhMan. **B)** The effect of the temperature on the activity of rHhMan.

The optimal enzymatic activity of rHhMan occurred at pH 5.5, with 95% activity at pH 5.0 and 75% activity at pH 4.0, 6 and 7. The activity gradually decreased to 50% at pH 7.5 and 8 (Figure 3A). These results demonstrate that rHhMan remains stable under acidic pH conditions, which is in agreement with the physiological pH levels in the digestive tract of *H. hampei*, which lie between pH 4.5 and 5.2 [25]. Similarly, the intestinal pH in other coleoptera is between 4.5 and 5.5 [26]. Other mannanases from diverse fungi exhibit optimal activity at acidic pH, and mannanases from *Aspergillus niger, A. fumigatus* and *A. oryzae* are optimally active at pH 5.5, 4.5 and 4.5, respectively [10,27,28].

The maximum activity of rHhMan occurred at 30°C, and the activity was reduced to approximately 70% between 10 and 20°C. The activity decreased substantially at high temperatures. At 40°C, 60% of the activity was conserved, while only 30% activity remained at 50°C (Figure 3B). These findings coincide with the reported activity levels in the digestive tracts of insects, with the highest activity between 30 and 40°C and a rapid decrease in the maximum activity at higher temperatures [26].

### Hydrolytic properties

**Figure 4:**
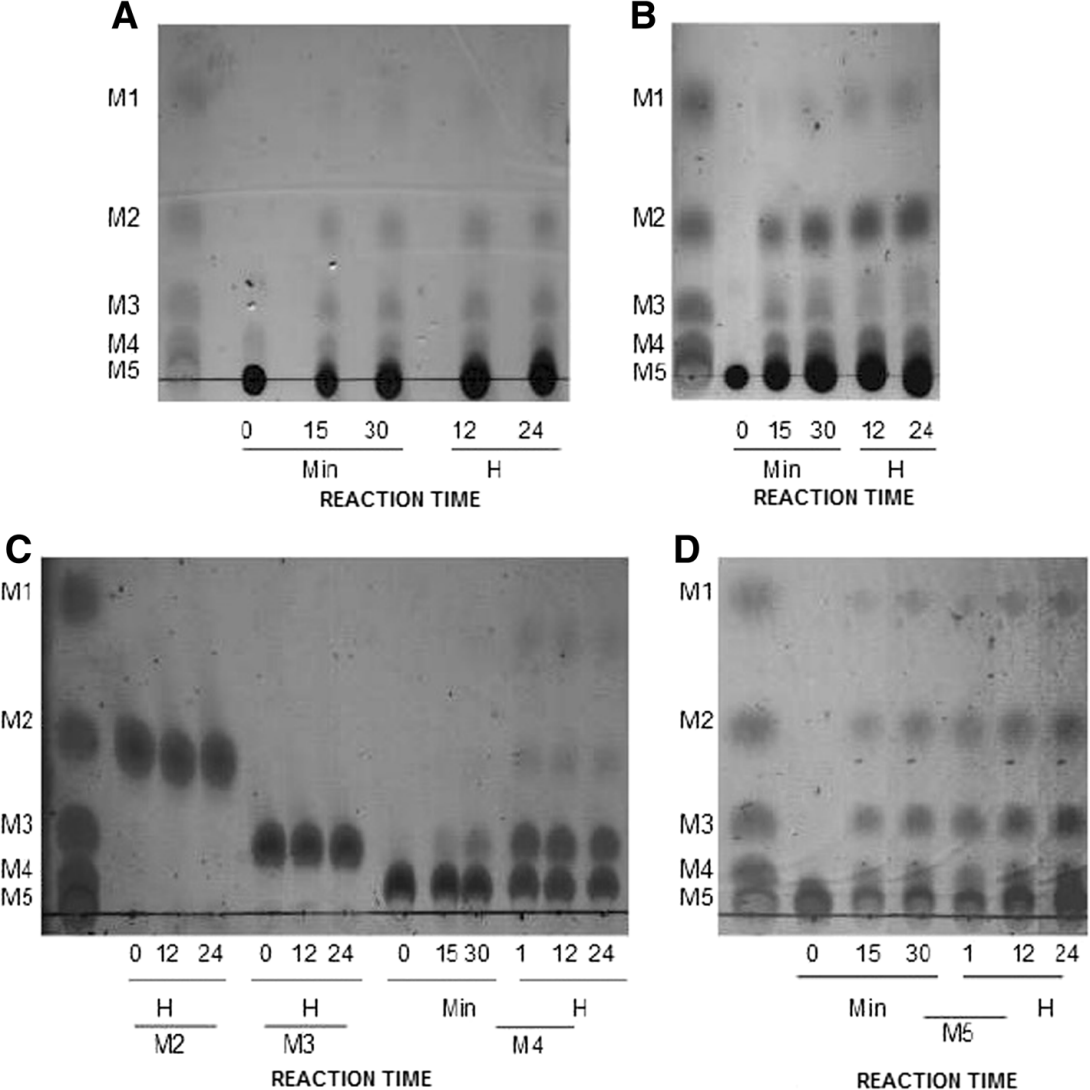
**Thin layer chromatography (tlc) of the hydrolytic products generated by rhhman on Mannans and Manno-oligosaccharides.** The reactions in the substrates respectives in 0,1 M sodium citrate buffer (pH 5.5) with 0,4 μg of rHhMan were incubated at 30°C for 24 hours. 2 μl of each reaction were analized by TLC. The substrates were **A)** Guar gum, **B)** Locust vean, **C)** Manno- oligosaccharides: mannobiose, mannotriose and mannotetraose and **D)** Manno-oligosaccharides: Mannopentose. The incubation time was shown (minutes and hours). The standar was a mix of mannose and manno- oligosaccharides where M1 is mannose; M2, mannobiose; M3, mannotriose; M4, mannotetraose y M5 mannopentose.

Purified rHhMan protein can hydrolyze high-molecular weight polysaccharides with β-1,4-glycosidic bonds in their structure. Figure 4A-B shows that the main by products of the hydrolysis of both guar and locust bean gum are mannose and manno-oligosaccharides of different sizes (mannobiose, mannotriose and mannotetraose) after a 15-min reaction. Similarly, other *Bacillus sp.* mannanases hydrolyze different substrates with the same structural characteristics of the aforementioned galactomannans [9]. In those reactions, the hydrolysis by products obtained can be used as food additives because of the prebiotic and anti-obesity effects that MOS have in different regions of the digestive tract [29].

The mode of action of rHhMan was analyzed by thin layer chromatography (TLC) with different manno-oligosaccharides (Figure 4C). The enzyme cannot hydrolyze mannobiose and mannotriose in a 24-hr incubation. However, mannotetraose hydrolysis produces mannose, mannobiose and mannotriose. This results suggest that probably it is a process that include a transglycosylation reaction in the mechanism that allows the enzyme to hydrolyze this manno-oligosaccharide [14,27]. In these types of enzymes, mannans must have a degree of polymerization of at least 4 units to achieve significant hydrolysis, as occurs with mannotetraose [30].

Additionally the knowledge of hydrolytic properties of this enzyme and the methodology described has been used to evaluate five analogues that mimic the HhMan substrate and function as inhibitors of the enzyme. The compounds were evaluated by TLC and enzymatic inhibiton test. One of the tested compounds, the 4-nitrophenyl-thio-β-D-mannopyranoside was identified as an inhibitor of HhMan. Thus, this strategy is a starting point for the development of new molecules to be used in pest control strategies to reduce the serious damage caused by the coffee berry borer during coffee cultivation [2].

### Kinetic parameters

**Figure 5:**
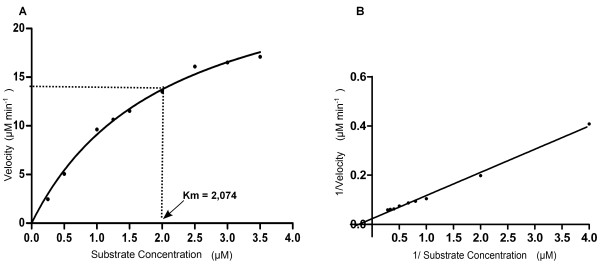
**Michaelis- menten and lineweaver- burk plots of rHhMan using guar gum as substrate. A)** Michaelis-Menten plot. **B)** Lineweaver-Burk plot. The measures were performed in optimum pH and temperature conditions.

rHhMan displayed Michaelis-Menten reaction behavior in guar gum substrate assays, as shown in Figure 5. This polysaccharide has a mannose:galactose ratio of 1.6:1 and, therefore, has a higher content of galactose than other polysaccharides in which galactose is substituted by other monosaccharides, such as locust bean gum (4:1) and carob gum (3.76:1). Our results indicate that the affinity of rHhMan for the guar gum substrate (K_m_= 2.074 mg ml^-1^) is similar to that reported for other mannanases for locust bean gum (K_m_=2.0 mg ml^-1^) and carob gum (K_m_= 2.2 mg ml^-1^) substrates. These data suggest that enzymatic activity is not affected by the presence of side chains such as galactose residues. In addition, rHhMan displays a higher affinity for guar gum than the mannanases of *Aspergillus niger* and *Bacillus subtilis* WY34 (K_m_= 7.7 mg ml^-1^ and 27.4 mg ml^-1^, respectively) [9,17].

The catalytic rate, k_cat_, for rHhMan is 50.87 s^-1^, which is greater than that of the mannanase of *Bacillus licheniformis*, which has a lower catalytic potential for glucomannan substrates (k_cat_= 21.00 s^-1^), locust bean gum (k_cat_= 31,20 s^-1^) and D-mannan (k_cat_= 18.2 s^-1^) [31].

## Conclusions

This study is the first report on an insect endo-1,4-β-mannanase expressed in *Pichia pastoris*. This enzyme has a molecular weight of 35.5 kDa without its native signaling sequence, belongs to the glycosyl hydrolase GH5 family and contains the eight strictly conserved residues found in proteins within this family. The enzyme exhibits optimal activity under conditions similar to those of the digestive tract of the coffee berry borer. The rHhMan protein can hydrolyze different galactomannans and manno-oligosaccharides that can be used in the pharmaceutical and food industries because of their beneficial effects on human health. Coffee beans are the only food source for coffee berry borer insects, and the role that this enzyme performs in the utilization of galactomannan, the main polysaccharide found in coffee beans, makes these results a good starting point for designing rHhMan inhibitors. According with previous studies made for evaluate synthetic inhibitors of the enzyme, the results of this investigations may be used in the development of new molecules that could be used in pest control strategies that would help reduce the economic losses resulting from this insect.

## Methods

### Endo-1,4-β-mannanase (HhMan) protein sequence analysis

The amino acid sequence of HhMan available (GenBank Accession Number ADF22325.1) was analyzed by multiple sequence alignment comparisons with BLAST (Basic Local Alignment Search Tool) and CLUSTALW, respectively [32,33]. Secondary structure prediction was performed with the PSIPRED server [34], and queries on protein family and domain information were performed with the Pfam database [35].

### Cloning, expression of the endo-1,4-β-mannanase (*HhMan*) gene and purification of the recombinant protein (rHhMan)

The cloning and heterologous expression of the *P. pastoris* system as well as the purification of the recombinant protein were performed under the methodology described previously [1]. The HhMan gene was amplified without its native signaling sequence with the primers Pp_Man_Fw (5′-CCG*CTCGAG*AAAAGAGTACCCGGATTCACGGTTTC-3′) and Pp_Man_Rv (5′-TGC*TCTAGA*CCATTGAATATTGAACAGATTG -3′). Pp_Man_Fw includes a *XhoI* restriction site, and Pp_Man_Rv includes the site for *XbaI* (italics).

### SDS-PAGE and zymography analysis

An aliquot of the purified recombinant protein and the commercial enzyme: Rohalase®GMP used like a positive control of mannanase activity were analyzed by SDS- PAGE (12%) as described by Laemmli [36]. The bands were visualized by Coomassie Blue R-250 staining. A low-range molecular weight standard (BIORAD, Hercules, California, USA) was used as a reference marker.

Another aliquot of protein and the positive control were used to assay enzymatic activity in a native PAGE, containing 1% (w/v) guar gum (Sigma, St Louis, Missouri, USA) as the substrate; activity was evaluated with 1, 1.5 and 2 μg of rHhMan, at 4°C. To evaluate enzyme activity, the gels were incubated in 0.1 M sodium citrate buffer (pH 5.5) for 20 min at 30°C. Enzymatic activity was visualized by Red Congo staining as described previously [1].

### Enzymatic assays

The activity of the rHhMan enzyme was determined by the 3,5-dinitrosalicylic acid (DNS) method [37]. The substrate, guar gum (1%), was dissolved in 500 μl of 0.1 M sodium citrate buffer (pH 5.5) and incubated with 0.05 μg of rHhMan enzyme at 30°C for 30 min. Rohalase®GMP was used like a positive control of mannanase activity. The amount of reducing sugars released during the reaction was measured by mixing 5 μl of the enzymatic reaction and 5 μl of DNS solution, according to the methodology described by Padilla [1]. One unit of endo-1,4-β-mannanase activity is defined as the amount of enzyme required to release 1 μmol of reducing sugar per minute under the experimental conditions described with D-mannose as the standard substrate.

### Effect of pH and temperature on enzymatic activity

The optimal pH for rHhMan activity was determined over a pH range of 3.5 to 8.0 under standard assay conditions with two buffering systems: 0.1 M sodium citrate (pH 3.0 – 6.0) and 0.1 M potassium phosphate (pH 6.0 – 8.0).

The effect of temperature on rHhMan activity was measured by incubating 0.05 μg of enzyme with 1% guar gum substrate in 0.1 M sodium citrate buffer (pH 5.5) at different temperatures between 10 and 60°C. The enzymatic activity was evaluated under standard assay conditions.

### Hydrolytic properties

Fractions of 0.4 μg rHhMan were added to 0.5% guar gum and locust bean gum solutions (Sigma, St Louis, Missouri, USA) in 0.1 M sodium citrate buffer (pH 5.5). Reactions were incubated at 30°C for 24 hr. Aliquots were collected at 0, 15, and 30 min time points as well as at 12 and 24 hr, and they were then heated at 100°C for 5 min. Hydrolysis byproducts were separated on 60F 254 silica plates (Merck, Darmstadt, Germany) with chloroform: ethyl acetate: n-propanol: water (0.2:1:1.5:0.5 v/v) and detected by sulfuric acid aspersion in 5% ethanol, followed by heating at 100°C for 5 min.

To determine the mode of action of the enzyme, 0.4 μg of rHhMan was added to 25 mM mannobiose, mannotriose and mannotetraose mannooligosaccharide solutions in 0.1 M sodium citrate buffer (pH 5.5). The reactions were incubated at 30°C for 24 hr, and aliquots were collected at different time points and heated at 100°C for 5 min. The hydrolysis products were separated on silica plates as previously described. A mannooligosaccharide mix of mannose, mannobiose, mannotriose, mannotetraose and mannopentose (Megazyme, Co., Wicklow, Ireland) was used as a standard.

### Kinetic parameters

To determine the kinetic parameters K_m_ and V_max_, guar gum substrate was used in a concentration range of 2.5 to 35 mg ml^-1^ in 0.1 M sodium citrate buffer (pH 5.5). The reaction velocity was determined in triplicate for each substrate concentration. The data were fitted to a nonlinear regression model of the Michaelis-Menten equation with Prism software (GraphPad Software, San Diego, California, USA).

## Competing interests

The authors declare that they have no competing interests.

## Authors’ contributions

CAG and JVO: Performed the identification, amplification and cloning of HhMan as well as the subcloning in a *Pichia* vector and expression. CAG: purification and biochemical characterization of HhMan, performed the HhMan enzymatic assays and drafted the manuscript. PGS: Helped in enzymatic assays design. RAZ: Conceived the study and participated in its design, coordination and helped draft the manuscript. All authors read and approved the final version of this manuscript.

## References

[B1] Padilla-Hurtado B Florez-Ramos C Aguilera-Gálvez C Medina-Olaya J Ramirez-Sanjuan A Rubio-Gomez J Acuna-Zornosa R Cloning and expression of an endo-1,4-beta-xylanase from the coffee berry borer. Hypothenemus hampei BMC Res Notes 2012 5 1 23 10.1186/1756-0500-5-23 22233686 PMC3283504

[B2] Aguilera-Gálvez C Gutierrez- Sanchez P Acuña-Zornosa R Modelado molecular e interacción enzima-ligando de potenciales inhibidores de la endo-1,4-β-mananasa de la broca del café *Hypothenemus hampei* Boletin de Investigaciones Unisarc 2012 10 1 17 23

[B3] Moreira L Filho E An overview of mannan structure and mannan-degrading enzyme systems Appl Microbiol Biotechnol 2008 79 2 165 178 10.1007/s00253-008-1423-4 18385995

[B4] Bustillo Pardey AE Una revisión sobre la broca del café, *Hypothenemus hampei* (Coleoptera: Curculionidae: Scolytinae), en Colombia Revista Colombiana de Entomología 2006 32 101 116

[B5] Redgwell R Fischer M Coffee carbohydrates Braz J Plant Physiol 2006 18 1 165 174 10.1590/S1677-04202006000100012

[B6] Jaramillo J Borgemeister C Baker P *Coffee berry borer Hypothenemus hampei* (Coleoptera: Curculionidae): searching for sustainable control strategies Bull Entomol Res 2006 96 03 223 233 10.1079/BER2006434 16768810

[B7] Yan X-X An X-M Gui L-L Liang D-C From structure to function: insights into the catalytic substrate specificity and thermostability displayed by *Bacillus subtilis* mannanase BCman J Mol Biol 2008 379 3 535 544 10.1016/j.jmb.2008.03.068 18455734

[B8] Yang P Li Y Wang Y Meng K Luo H Yuan T Bai Y Zhan Z Yao B A novel β-mannanase with high specific activity from *Bacillus circulans* CGMCC1554: gene cloning, expression and enzymatic characterization Appl Biochem Biotechnol 2009 159 1 85 94 10.1007/s12010-008-8364-3 18820840

[B9] Jiang Z Wei Y Li D Li L Chai P Kusakabe I High-level production, purification and characterization of a thermostable β-mannanase from the newly isolated *Bacillus subtilis* WY34 Carbohydr Polym 2006 66 1 88 96 10.1016/j.carbpol.2006.02.030

[B10] Naganagouda K Salimath PV Mulimani VH Purification and characterization of endo-beta-1,4 mannanase from *Aspergillus niger* gr for application in food processing industry J Microbiol Biotechnol 2009 19 10 1184 1190 19884778

[B11] Johnson KG Ross NW Enzymic properties of β-mannanase from *Polyporus versicolor* Enzyme Microb Technol 1990 12 12 960 964 10.1016/0141-0229(90)90117-9

[B12] Malheiros Ferreira H Ximenes Ferreira Filho E Purification and characterization of a β-mannanase from *Trichoderma harzianum* strain T4 Carbohydr Polym 2004 57 1 23 29 10.1016/j.carbpol.2004.02.010

[B13] Marraccini P Rogers WJ Allard C André M-L Caillet V Lacoste N Lausanne F Michaux S Molecular and biochemical characterization of endo-ß-mannanases from germinating coffee (*Coffea arabica*) grains Planta 2001 213 2 296 308 10.1007/s004250100541 11469596

[B14] Xu B Hägglund P Stålbrand H Janson J-C Endo-β-1,4-Mannanases from blue mussel, *Mytilus edulis*: purification, characterization, and mode of action J Biotechnol 2002 92 3 267 277 10.1016/S0168-1656(01)00367-4 11689251

[B15] Ootsuka S Saga N Suzuki K-i Inoue A Ojima T Isolation and cloning of an endo-β-1,4-mannanase from Pacific abalone *Haliotis discus hannai* J Biotechnol 2006 125 2 269 280 10.1016/j.jbiotec.2006.03.008 16621092

[B16] Cantarel BL Coutinho PM Rancurel C Bernard T Lombard V Henrissat B The carbohydrate-active enzymes database (CAZy): an expert resource for glycogenomics Nucleic Acids Res 2009 37 suppl 1 D233 D238 18838391 10.1093/nar/gkn663 PMC2686590

[B17] Bien-Cuong D Thi-Thu D Berrin J-G Haltrich D Kim-Anh T Sigoillot J-C Yamabhai M Cloning, expression in *Pichia pastoris* and characterization of a thermostable GH5 mannan endo-1,4-β-mannosidase from *Aspergillus niger* BK01 Microb Cell Fact 2009 8 1 1 12 10.1186/1475-2859-8-1 19912637 PMC2780388

[B18] Baird SD Hefford MA Johnson DA Sung WL Yaguchi M Seligy VL The Glu residue in the conserved Asn-Glu-Pro sequence of two highly divergent endo-β-1,4-glucanases is essential for enzymatic activity Biochem Biophys Res Commun 1990 169 3 1035 1039 10.1016/0006-291X(90)91998-8 2363713

[B19] Guiseppi A Cami B Aymeric JL Ball G Creuzet N Homology between endoglucanase Z of *Erwinia chrysanthemi* and endoglucanases of *Bacillus subtilis* and alkalophilic *Bacillus* Mol Microbiol 1988 2 1 159 164 10.1111/j.1365-2958.1988.tb00017.x 2835589

[B20] Macarron R van Beeumen J Henrissat B de la Mata I Claeyssens M Identification of an essential glutamate residue in the active site of endoglucanase III from *Trichoderma reesei* FEBS Lett 1993 316 2 137 140 10.1016/0014-5793(93)81202-B 8093602

[B21] Gilbert HJ Stålbrand H Brumer H How the walls come crumbling down: recent structural biochemistry of plant polysaccharide degradation Curr Opin Plant Biol 2008 11 3 338 348 10.1016/j.pbi.2008.03.004 18430603

[B22] Schröder R Wegrzyn T Sharma N Atkinson R LeMAN4 endo-β-mannanase from ripe tomato fruit can act as a mannan transglycosylase or hydrolase Planta 2006 224 5 1091 1102 10.1007/s00425-006-0286-0 16649044

[B23] Zahura UA Rahman MM Inoue A Tanaka H Ojima T An endo-β-1,4-mannanase, AkMan, from the common sea hare *Aplysia kurodai* Comp Biochem Physiol, Part B: Biochem Mol Biol 2010 157 1 137 143 10.1016/j.cbpb.2010.05.012 20639136

[B24] Song JM Nam K-W Kang SG Kim C-G Kwon S-T Lee Y-H Molecular cloning and characterization of a novel cold-active β-1,4-d-mannanase from the Antarctic springtail, *Cryptopygus antarcticus* Comp Biochem Physiol, Part B: Biochem Mol Biol 2008 151 1 32 40 10.1016/j.cbpb.2008.05.005 18579426

[B25] Valencia A Bustillo AE Ossa GE Chrispeels MJ α-Amylases of the coffee berry borer (*Hypothenemus hampei*) and their inhibition by two plant amylase inhibitors Insect Biochem Mol Biol 2000 30 3 207 213 10.1016/S0965-1748(99)00115-0 10732988

[B26] Silva EM Valencia A Grossi-de-Sá MF Rocha TL Freire É de Paula JE Espindola LS Inhibitory action of Cerrado plants against mammalian and insect α-amylases Pestic Biochem Phys 2009 95 3 141 146 10.1016/j.pestbp.2009.08.003

[B27] Puchart V Vršanská M Svoboda P Pohl J Ogel ZB Biely P Purification and characterization of two forms of endo-β-1,4-mannanase from a thermotolerant fungus, *Aspergillus fumigatus* IMI 385708 (formerly *Thermomyces lanuginosus* IMI 158749) Biochimica et Biophysica Acta (BBA) - General Subjects 2004 1674 3 239 250 10.1016/j.bbagen.2004.06.022 15541293

[B28] Regalado C García-Almendárez BE Venegas-Barrera LM Téllez-Jurado A Rodríguez-Serrano G Huerta-Ochoa S Whitaker JR Production, partial purification and properties of β-mannanases obtained by solid substrate fermentation of spent soluble coffee wastes and copra paste using *Aspergillus oryzae* and *Aspergillus niger* J Sci Food Agric 2000 80 9 1343 1350 10.1002/1097-0010(200007)80:9<1343::AID-JSFA651>3.0.CO;2-#

[B29] Smith DL Nagy TR Wilson LS Dong S Barnes S Allison DB The effect of mannan oligosaccharide supplementation on body weight gain and fat accrual in C57Bl/6J mice Obesity 2010 18 5 995 999 10.1038/oby.2009.308 19798073 PMC2940117

[B30] Ademark P Varga A Medve J Harjunpää V Torbjörn D Tjerneld F Stålbrand H Softwood hemicellulose-degrading enzymes from *Aspergillus niger*: Purification and properties of a β-mannanase J Biotechnol 1998 63 3 199 210 10.1016/S0168-1656(98)00086-8 9803534

[B31] Songsiriritthigul C Buranabanyat B Haltrich D Yamabhai M Efficient recombinant expression and secretion of a thermostable GH26 mannan endo-1,4-beta-mannosidase from *Bacillus licheniformis* in *Escherichia coli* Microb Cell Fact 2010 9 1 20 10.1186/1475-2859-9-20 20380743 PMC2868798

[B32] Altschul SF Gish W Miller W Myers EW Lipman DJ Basic local alignment search tool J Mol Biol 1990 215 3 403 410 2231712 10.1016/S0022-2836(05)80360-2

[B33] Thompson JD Gibson TJ Plewniak F Jeanmougin F Higgins DG The CLUSTAL_X Windows Interface: Flexible Strategies for Multiple Sequence Alignment Aided by Quality Analysis Tools Nucleic Acids Res 1997 25 24 4876 4882 10.1093/nar/25.24.4876 9396791 PMC147148

[B34] Buchan DWA Ward SM Lobley AE Nugent TCO Bryson K Jones DT Protein annotation and modelling servers at University College London Nucleic Acids Res 2010 38 suppl 2 W563 W568 20507913 10.1093/nar/gkq427 PMC2896093

[B35] Finn RD Mistry J Schuster-Böckler B Griffiths-Jones S Hollich V Lassmann T Moxon S Marshall M Khanna A Durbin R Pfam: clans, web tools and services Nucleic Acids Res 2006 34 suppl 1 D247 D251 16381856 10.1093/nar/gkj149 PMC1347511

[B36] Laemmli U Cleavage of structural proteins during the assembly of the head of bacteriophage T4 Nature 1970 227 680 685 10.1038/227680a0 5432063

[B37] Peter B Colowick SP, Kaplan NO Amylases, α and β Methods in Enzymology 1955 1 New York: Academic Press 149 158

